# A Treatise on Those Diseases of the Brain and Nervous System, Usually Called Mental

**Published:** 1834-01-01

**Authors:** 


A Treatise on those Disorders of the Brain and Nervous System, which are usually considered and called Mental. By *David Uwins*, M.D. Octavo, pp. 233. Renshaw and Rush. Sept. 1833.

To publish another work on insanity, in the present depressed state of the *book-trade*, is little less than an indication—or, at all events, a premonitory symptom of the disease itself. Our worthy author indeed acknowledges, near the end of the work, that we are all more or less insane—" it follows that the difference between sanity and acknowledged insanity, is rather a difference *in degree* than in kind"—a sentiment in which we cannot fully agree with Dr. Uwins !*

Dr. Uwins opens his case in the following manner:

" Circumstanced as is the author of the present treatise in reference to cases of mental aberration, having constantly under his care every grade and every shape of deranged intellect; now being called to visit in the Asylum to which he is professionally attached, the horrible convulsions constituting an epileptic paroxysm, now the circumstances and consequences of an apoplectic seizure; on this day being required to investigate a malady, the features of which may scarcely be visible without painful scrutiny; on another day being placed almost in personal danger by the sufficiently palpable and violent ebullitions of maniacal fury; at one moment having to trace the oftentimes faint line of demarcation between inflammation of the brain, and states simulating it, at the next to aim at ascertaining whether visceral or vascular conditions are the sources, or only the incidents of derangement; being, moreover in many cases, called on (and sometimes for judicial purposes) to decide the very difficult, but very momentous question, whether acts have been the result of controulable impulse or delusive excitation; it may not be charged upon an individual thus engaged, that he is incompetent to write on insanity, on the ground that what he writes must rather be the closet coinage of his own brain, than the fruits of actual and practical observation." 2.

No one will question Dr. U.'s competency to write on insanity or on any other professional subject; but there are some who will not praise the style or the taste of the foregoing passage. This is of little consequence, but the inattention to strict arrangement, and the desultory manner in which the subjects are treated, render it totally impossible for us to give an analysis of the work. We must therefore throw out our hook at random, and catch what we can.

Dr. U. observes that "erroneous *conception*" constitutes the main ingre-

---

* While writing these lines we see that Dr. Uwins stated this opinion of universal insanity at the Old Bailey, in the case of Elizabeth Wrattan. We apprehend that such a doctrine should not be publicly stated, first, because it is not correct, and secondly, because it is highly impolitic, and would defeat the ends of justice. If all are insane, as the judge remarked, none are sane—and none, at that rate, were accountable agents. If the Doctor had said that all were, more or less, *unwise*, he would have been nearer the mark. The jury gave it against the Doctor in this trial.

dient of positive madness—the misconception being always more or less engendered by false *perception.*

"I have had an interview with a young gentleman of first rate accomplishments, and full of intellectual vigour and propriety, who, immediately you mention the word elephant, whether by design or accident, whether in combination or by itself, is suddenly seized with a species of horrific spasm; and while the impression lasts, is, to all intents and purposes, a madman." 6.

We know a gentleman whose life is rendered wretched by the number 3. Whenever that unlucky number comes across him in any conspicuous character, he is overcome with a fit of horrors, and continues so for several days, though his reason tells him it is an illusion. His feelings, however, will not permit him to shake off the illusion, till by some means or other he contrives to break the spell, when he is as well as ever. Thus he fancied a watch which he saw hanging up in a pawnbroker's window, and purchased it. Some time afterwards, while winding it up, he saw to his astonishment that the number of it was 333; and to add to the misery, the number of the pawnbroker's house was 33—while, to crown the infernal catalogues of *threes,* he remembered that he paid *three* guineas for the watch!! Ridiculous as this chain of coincidences may appear, it cost the poor gentleman more than six months of mental suffering, during which he pined away to a skeleton, and was, at length, obliged to travel three or four hundred miles to London, in order to find out the pawnbroker, to whom he returned the watch for two guineas. From that instant, the spell was broken, and he was himself again!

After a short review of the phrenological system, as elucidatory of insanity, the Doctor leaves the point undecided—balancing so adroitly between the phrenologists and anti-phrenologists, that it is difficult to say what is his own opinion, if, in reality, he has one. But he winds up the first chapter with the following conclusion, namely, "that mere nervousness, as it is vulgarly named, and insanity, as it is almost as vaguely assumed, are absolutely identical." We suspect that some of the Doctor's nervous patients would not be overwell pleased if he were to broach this doctrine in their presence. We beg leave to suggest to the worthy Doctor the propriety of keeping such sentiments to himself; for surely a great proportion of his patients will be shy of having his carriage at their doors, seeing that, by his own shewing, *all* his patients are more or less insane. Let the Doctor look to this in time!

The second chapter opens with "definition"—"essentials." Considering nervousness as a degree of insanity, Dr. U. justly observes that those brief summings up, entitled definitions, are no easy matters. Notwithstanding this difficulty, our author jumps at a very laconic definition, contained in a single word—insanity is neither more nor less than "MISCONCEPTION." The meaning of this word, in Johnson's Dictionary, is "false opinion." So every person who forms a false opinion is insane! This is certainly in keeping with Dr. Uwins' opinion, whether that opinion be true or false. The illustration which Johnson gives of misconception, is not very inapplicable to the doctrines of our worthy author. "It cannot be that our knowledge should be other than a heap of *misconception* and error."—*Glanville.* Dr. Uwins, however, treats us to the various definitions of insanity given by Darwin, Brown, Beddoes, Watts, Mead, Haslam, Shakespeare, Arnold,

Conolly, and many others, though we know not for what purpose, since "misconception" is the most easily remembered of any. Indeed as we are *all* insane, we think that OMNIBUS is just as good a definition for insanity, as any given—by Dr. Uwins himself.

To the kinds and shapes of insanity, as mania, melancholia, monomania, hypochondriasis, dementia, &c., our author attaches little importance.

"*Melancholia*—why we are all melancholic at times from misconception. *Mania*, who is there that has not been at one period or another irritated beyond measure by misapprehension? *Monomania*, or misconception on one point. What light do we throw upon this feeling by giving it a hard name! Of *hypochondriasis* we have plenty of instances in the present day, much short of madhouse dreamers, since the stomach has been considered every thing, and every thing the stomach. Even *Idiotcy* may be monoidiotism, as seems to have been the case in the Baxter instance; and we are all more or less idiotic, if the term implies congenital want of power." 32.

The third chapter is devoted to the progress of symptoms—and to general characteristics. As may be supposed, Dr. Uwins considers insanity as depending on bodily disorder—and there we agree with him. He does not confine the corporeal cause to any particular part of the body, but places it in the head, chest, liver, stomach, and any or every spot of the corporeal fabric. In this chapter there are some loose and scattered hints and observations which are worth attending to. Dr. U. remarks that every individual case may be looked upon as a fresh study—bringing with it peculiar incidents and special features.—Hence a history of lunacy can only be regarded as a collection of the more general and prominent circumstances by which it is characterized.

It has been thought that the walk of insane persons is peculiar. If the space be confined, the maniac will shew an impetuosity in his movements. Dr. Uwins, and others, notice the offensive breath peculiar to deranged individuals. It is not always from derangement of the stomach. Passions of the mind will immediately produce these changes in the pulmonary exhalations, without any apparent disorder of the digestive organs.

"The talkative lunatic is remarkable for the repetition of a word that may have been accidentally used by himself, or expressed by another; and such repetition is, for the most part, still more persisted in, despite of endeavours of bystanders to supersede it, should it fall into a sort of rhythm with other parts of a sentence. On the other hand, what has been called by Shakespeare rewording, can seldom be accomplished by an individual whose ideas are jumbled together insanely. This difficulty in distinctly repeating a proposition, ought to be recollected by all who are professionally called on to decide mental conditions, as it is extremely significant of derangement. It was alluded to particularly, in a paper read before the College of Physicians, some time since, by the President." 43.

There are so many people, however, whose ideas are "jumbled together," and who find difficulty in repeating a proposition, or any thing else correctly, that we should not depend much upon this criterion.

Maniacs are so taken up with the intensity of their feelings as to be often insensible to the lapse of time. The affecting instance recorded by Mr. Hill, is a remarkable illustration.

"A gentleman on the point of marriage left his intended bride for a short time; he usually travelled in the stage coach to the place of her abode; the last journey he took from her was the last of his life. Anxiously expecting his return, she went to meet the vehicle. An old friend announced to her the death of her lover. She uttered an involuntary scream, and piteous exclamation, 'he is dead!' From this fatal moment, *for fifty years*, has this unfortunate female daily, in all seasons, traversed the distance of a few miles, to the spot where she expected her future husband to alight from the coach, uttering in a plaintive tone, 'he is not come yet—I will return to-morrow.'" 44.

Several other traits of insanity are delineated by our author, all shewing that he is well conversant with this species of human calamity.

In the fourth chapter, Dr. Uwins traces the sources of insanity—insisting, with much justice, that—"the sources and resources of refinement are, in a great measure, the sources of insanity." We find our author a stanch advocate of the doctrines of Cobbett, that the diffusion of knowledge is a great evil—and a great cause of insanity.

"Science may boast of her steam rapidity. Railway contrivances may come almost to annihilate the notion of time and distance. The arts of luxury may pour out their enchantments, and philosophy and mercantile industry join to convey them to the remotest villages; but even allowing that there is a benefit to society in all this, how certain and how great is its evil among the peasantry. Were I to say that, even in my time, the countryman has been a much healthier and happier, because a more ignorant man, than he is at present, I might be called an enemy to improvement—a conservative of feudal barbarity. I feel, however, that the charge would be ill-founded; and, beside, I am only required to state facts physiologically, and in reference to our present inquiry; and most certain it is, that they infer too largely respecting the benefits of science—sometimes falsely so called—who calculate upon its unalloyed blessings. Dr. Willis used to say that he owed a great proportion of his patients to the importation of China tea into Britain. In this assertion he might be correct in one sense, but not in another. It is not the mere abstract poison of tea which deteriorates the nervous system,—though there is something even in this,—*but it is the accompaniments which tea brings with it that do the greatest part of the mischief.* Pianos, parasols, Edinburgh Reviews, and Paris-going desires, are now found among a class of persons who formerly thought these things belonged to a different race; these are the true sources of nervousness and mental ailments, and not merely this or that specific article of food or drink." 51.

In the foregoing passage the more profound pathologist will see that Dr. Uwins mistakes concomitants for causes. The pianos, the parasols, and the Edinburgh Reviews, have little to do, as *causes* of insanity. They are only amusements and implements which high civilization requires—or determines to employ, and no more. In the following passage this mistake is still more conspicuous.

"It is a curious fact, and it being mentioned here may serve to strengthen my assumption, that the multiplication of rules about diet and regimen brings with it an increase of the very evil that is so anxiously sought to be avoided. When did dyspepsia prevail so hugely as it does at this moment, when we have treatise upon treatise, and precept upon precept on stomach complaints. Who ever heard of such a numerous host of heart affections as now exist, now that the very vulgar talk largely and learnedly about valves and ventricles, and functions and organs? Do not teeth disorders increase in number with the multiplication of dentists, even of science and principle? and are not female complaints manifestly more numerous and complicated, now that there is an obstetrician in every street?" 52.

In the first place, it is to be remembered that Dr. Uwins has written a very learned treatise on diet and indigestion,—consequently he has his share of the dyspeptic sins to answer for, in company with Abernethy, Phillip, Paris, Abercrombie, and many others. But does Dr. Uwins seriously believe that stomach doctors have caused the increase of stomach disorders—that Corvisart, Laennec, Forbes, Hope, &c. have spread diseases of the heart—that dentists have multiplied the tooth-ache—and that Sir Charles Clarke, Dr. Merriman, Dr. Granville, Dr. Davies, Drs. Ley and Lee, Dr. Locock, Professor Davis, &c. &c. have increased leucorrhœa, amennorrhœa, abortions, floodings, cancer uteri, and the various uterine maladies to which our wives, daughters, and elderly aunts are subjected? This doctrine is precisely on a par with the etiology of the Goodwin Sands—which sands having appeared soon after the erection of Tenterden Steeple—were, on the sound argument of "post hoc ergo propter hoc," very naturally attributed to that useless appendage to Tenterden Church.

Dr. Uwins touches on the subject of religion, as an occasional cause of insanity, with considerable caution. The arguments pro and con might be compressed into a nut-shell. It is not religion, but fanaticism and superstition that cause insanity. When we contemplate the gloomy, absurd, and frightful doctrines that are held forth by a hundred different sects, each "dealing damnation" on the tenets of the other, and consider the weakness of the minds on which these conflicting creeds are impressed, the wonder is that—Dr. Uwins is not right in his "idolum specus"—and that all men are not mad, according to the Doctor's theory!

Dram-drinking and opium-eating are touched upon by Dr. Uwins with considerable effort at impressive writing—but not always with the desired success. The following is a specimen.

"It has been generally assumed, that insanity is more common in England than elsewhere; but a census, taken from the time that the continent has been shaken by political and religious commotions, while we have been in comparative tranquillity, tells a different tale. The French Revolution, while it overthrew one monarch, created many. 'Nay, the madhouses of France at this time were peopled with gods as well as kings. Three Louis XVI.'s were seen together disputing one another's pretensions. There were, besides, several King's of Corsica and other countries; there were sovereigns of the world, a Jesus Christ, a Mahomet, so many deities as to render it necessary to distinguish them by the place they came from, as the god of Lyons, the god of the Gironde.'" 58.

To the flowing eloquence of the late Dr. Reid, our author is not a little indebted; and it would have been as well to have quoted his name a little more frequently, when making use of his sentiments.

Intense study is ranged among causes of insanity—and the following quotation is the whole of what is said on this point of etiology.

"Intense study has been ranged among the sources of insanity—and with propriety. Without being desirous of checking the youthful aspirant after college honours, I cannot but admit, that when 'hard reading' is carried beyond the physical powers, it is, like the *Vis consili expers* of the poet, calculated to destroy its own design. What would some of our pale-faced voluptuaries in study say to the advice of a lady, who, when writing to her son at the university, tells him, 'he must eat and drink like a ploughman, or he will do no good with his books ?' Certain it is, that if we do not walk, and eat, and sleep well, while at College, we gain our honours at a dear rate, and shall hold them upon an insecure tenure." 60.

As the majority of our youths eat, drink, and sleep well at College, we have not much to fear from this item in the long list of causation. Masturbation is alluded to in delicate terms—and its consequence, real or supposed impotency. In the influence of the moon, as productive or aggravative of lunacy, Dr. Uwins has not much faith; though he admits that atmospheric changes exert a great agency on lunatics. He is often able, "to predict with accuracy what will be the general condition of the Peckham inmates, in respect to perturbation and excitement, before he enters the house."

We are not inclined to agree with our author in the opinion that the aërial mutations in our atmosphere, so sudden and frequent, are the cause of "the nervousness and gloom which are said to be endemical in Britain." By correct reasoning, these frequent vicissitudes in the atmosphere should make Britons as fickle and variable as our French neighbours. But it is highly probable that moral causes have more to do with moral character, in this country, than the mere state of the atmosphere. Every body knows that the number of suicides in Paris is far greater, in proportion to the population, than in London—which cannot be from climate.

The fifth chapter is on "Pathological, or Proximate Causes." It may be very readily conceived that Dr. Uwins is able to throw little light on the proximate cause of such a disease as insanity. He admits that phrenology, "if correct in its applications, would go a considerable way towards explaining that irregularity of thought and act now supposed."

"But even were this doctrine admitted in all its required latitude, much would still remain inexplicable as to the mode and manner in which the phenomena of madness are produced. The organ of high-mindedness we will suppose to be at work—but what is the kind of change effected throughout its mass? If you answer it is excited, still the question recurs, what is the physical condition by which this excitation is constituted? We know that both brain and nerve are susceptible of the greatest change without any primary alteration, at least of an appreciable or discoverable extent, in the vascular part of our organization, and I hold that the phrenologist is in error, even upon his own principles, if he assume the excitement of an organ to be an inflammation of that organ." 67.

We do not know that the phrenologist maintains any such doctrine as that excitement is inflammation—and therefore the supposed *error* is a gratuitous assumption of the author himself. Some of Dr. Uwins' illustrations of irregular action in the brain are rather curious.

"We further see, that blood shall be conveyed in too large or too small a measure through the portal vein; the biliary secretion shall now flow freely and orderly through its appropriate conduits; now be sent into the stomach, *because* it is either formed in too large a quantity, or finds *a difficult transmission through its ducts*." 68.

How bile is thrown into the stomach by difficult transmission through the biliary ducts into the duodenum, would puzzle all the physiologists in Europe. How does the bile get into the stomach at all, but through its ducts? Would a total occlusion of the ducts throw all the bile into the stomach? It ought to do so, according to our author's explanation.

We agree with Dr. U. "that mania is not inflammation of the brain, necessarily or originally." He adduces the disease termed "delirium tremens," where the insanity is furious, and yet where opium proves the best remedy.

"In what is considered the low state, as opposed to maniacal flights, and which flights, by the way, often descend to lowness with the greatest rapidity, a morbid state of the liver and its functions is not, as above intimated, unfrequently met with, either as cause or consequence. Respecting the action of this viscus, as well as that of the kidneys, much information is wanting: I mean their actions in reference to their bearings on the animal economy, whether in a disordered or sound condition; it may be, that some states of brain and nerve derangement may grow out of, or at least have some alliance with a want of due quantity or quality in the blood that passes through the *vena-porta;* and in consequence a deficient transmission of venous blood may occasion a retention in the system of what nature designed to be excreted. Or an irregularity in the blood's distribution through the brain may interfere with the cerebral functions; and thus the derangement in bile secretion may be either cause or consequence of brain disorder." 73.

We shall pass over the remainder of this chapter, which thus terminates· "Our conclusion from the whole is the conclusion of Rasselas, in which nothing is concluded."

The sixth chapter, on prognosis, presents nothing remarkable. In the seventh, on prevention, Dr. Uwins seems to fear that he will be considered by some as being—"*bitten* by phrenology." Let the doctor take heart—it is a mere scratch—it will come to nothing. The following passage is bearing on the phrenological bite. We would almost expect that our author had been taking a lesson or two, *in tongues*, at Babel Chapel, Regent Street.

"On the day immediately preceding that on which I am now writing, (July 8, 1833,) I witnessed the public exhibition of two very extraordinary men; the cranial formation of which men exactly answers to the respective qualities of their mind, as exhibited from the pulpit; and I have very little, if any doubt, that these individuals, had they been differently circumstanced from what they have been, would at this moment have had their heads moulded with proportioned variety. The high imaginative organization of the one, and the combination of these tokens with that of the reasoning and perceptive indications of the other, need only be stated to some of my readers, and they will easily understand to whom I refer.

Who is there that does not estimate highly the grand points of character displayed by one of these persons, mixed, as they are, with so many humiliating vagaries? and who does not feel the value of cultivation, lopping, and pruning, could it be applied to push out those parts of his mental organization, which, from lying dormant, permit the other faculties to take such lofty flights and eccentric courses? There is, indeed, a grandeur in the mental circumstance altogether of the poetical preacher now referred to, which cannot fail of exciting the greatest respect, mingled, as the respect must be, with commiseration for his wanderings; and it is especially deplorable to feel that, without any deterioration of his genius, his perceptive and reasoning powers, had they been brought duly out, might have prevented him from becoming the ridicule of inferior men, who eagerly seize opportunities for running down, as it is expressively termed, those rich endowments, to the worth and value of which they are quite insensible." 97.

We confess that the "*rich endowments* and the fine *reasoning powers*" of the raving fanatic, or rather the transcendental lunatic to whom Dr. Uwins alludes, never excited in our minds, other than pity for the credulity and contempt for the absurdity of the Neophytes, as well as their high priest, in the temple in question.

Our author has introduced some judicious observations, in this chapter, on education, which we hope will be useful to general readers, by whom, indeed, the work will be chiefly patronized. His observations on religion too are sensible and deserving of perusal. But we must pass on to the eleventh chapter, on "the medical management of the insane."

Dr. Uwins observes that nothing could be more inconsistent than the reports made by medical men before the Commissioners of Inquiry, as to the curability of insanity. One lauded this, another that remedy. Emetics were to do every thing, according to the opinion of some—purgatives were almost the only medicines necessary, according to the opinion of others—and so on.

"Whence all this discrepancy and contradiction? In a great measure, I would be bold enough to say, in consequence of the subject being regarded in too empirical a light. Medical men have spoken of curing lunacy, as the vulgar talk of curing a cough. Indeed, while a specific cognomen is made to include so many varieties of degree, the question respecting the curable or incurable nature of insanity cannot well be put. Curable? most certainly it is, when originating from a known cause of a remedial nature, when connected with a bodily affection of an obvious and tangible kind, when taken early under proper cognizance, when happening to the comparatively young, when the constitution it attacks is good, and when the remedial measures are instituted with decision under the guidance of discernment. Incurable? undoubtedly, when it is a disorder of very old age, and comes upon an individual in that gradual and insidious manner, so faithfully delineated by Le Sage, in the concluding part of his inimitable work. When it is the consequence of a sabre or other wound occasioning an organic lesion of the brain, when it results from an inveterate apoplexy of a particular kind, when other diseases of the brain have produced an organic alteration in a portion of the brain mass, when it is connected with an incurable epilepsy, when the exciting cause has been so sudden and so intense that the whole system of thought and feeling is overturned, as it were, from its base, as in the case I have cited from Mr. Hill; or when the malady has run on from a functional into an unequivocally structural state, which is often the case in the course of a year; but sometimes it takes a longer, sometimes a shorter period, to effect this irremediable change." 190.

Dr. Uwins goes on to make some remarks on apoplexy, as a cause or premonitory symptom of insanity. It is not, however, the common or unequivocal apoplexy that he treats of, but an apoplectic condition or tendency, not very clearly defined. We shall give it in his own peculiar phraseology.

"It is not seldom that a species of chronic action shall have place in the vessels of the brain or its membranes, which shall last a considerable time, proceed to a considerable length, be the occasion of much uneasiness and irritation upon the patient's feelings, and at last produce the absolutely apoplectic condition of suspended sense and motion: this state sometimes terminating in aberrated rather than suspended perception, and then being more entitled to the name of insanity, according to the general acceptation of the word.

Now it is this condition of the brain which it behoves the pathologist and practitioner especially to recognize as a prelude to apoplexy or madness." 192.

Without identifying insanity with cerebral inflammation, Dr. Uwins acknowledges that a great proportion of our suicidal cases, and "a large number of mental aberration, owe their origin to the vascular, insidious, slow action in the brain and its membranes now supposed."

"This is especially the case in the insanity of those individuals who, by their professional calling and public functions, *stretch their brain power beyond its capacity of endurance*—and when we hear of the fate of such men as Romilly, Whitbread, and Londonderry, we should look upon these men as having been upon the brink of either apoplexy or madness, and only having been preserved from it by their own hands!" 192.

The line which we have marked in italics is a fair specimen of the peculiar and singularly uncouth phraseology which our author appears to take infinite pains to employ, though nothing can be more barbarous or grating to the ear. Dr. Uwins can write very well, if he pleases; but he evidently studies more to be rough than smooth in his language.

We extract the following piece of practical information from the work.

"Now, beyond bleeding and purging, I know nothing to which we cân attach much value in apoplexy; it is, however, of importance that, while we are decided and bold in cases of abolition of sense, occasioned by a rush of blood and inordinate fulness, with sthenic condition of body and brain, that we stay our hands from too much depletion, either in the apoplexy of hæmorrhage or that of serous debility. Indeed, this last is perhaps not of very common occurrence; but there is a chronic condition of brain, which may be viewed as in some measure allied to a serous state of the organ, and which is often to be met with in early life. Children are constantly under my inspection, whose brains, instead of going through the natural or healthy process of growth, become enlarged, and the infantine faculties are in corresponding hebetude; but which children you cannot pronounce to have positive hydrocephalus, even of the chronic kind; it should rather seem that there is a debility in the exhalent, or rather, supplying vessels, and a proportionate weakness in those vessels which take up part of the deposit, so that although growth or enlargement goes on, and is even increased unduly, the substance thus developing itself is not true and healthy brain mass. In these cases I have found abundant benefit to attend the use of exceedingly small doses of fox-glove. Two drops of the saturated tincture given three times a day are soon followed by a manifest improvement. The exhalent and absorbent vessels, or, if you please, the exhalent and absorbent power of the blood-vessels, is increased by these small doses of digitalis; the pulse becomes improved, the flesh more firm, and the brain in a better state. When with these appearances of the brain, a disordered condition of the mesenteric vessels displays itself by tumid knotty abdomen, and alvine discharges of an unhealthy appearance, I combine two or three grains of the *hydrarg. cum creta* with the night dose of fox-glove; but although some might be disposed to ascribe all the benefit to this last medicine, they would be in error in so doing, since I very often administer the other medicine alone, and with as much satisfactory result as can be expected to follow any curative plan applied to chronic maladies.

There is another state of things in which I have found—at least if my perceptions do not mislead me—this extraordinary drug to be administered with extraordinary success. Patients are constantly applying with complaints of feelings about the head, and throughout the whole frame, which produce an indescribable irritability; their tempers, they say, are soured they know not why; their thoughts are confused in a manner they cannot account for; they feel at times as if they were going to lose their senses. Now, if added to these statements of your patients you find a sort of knitting in the brow, a wiryness, as also a quickness about the pulse, and a febrile condition of the skin, with the secretions rather less and of a higher colour than *merely* nervousness gives rise to, it is fair to presume upon some such condition of the brain as that in the puerperal case alluded to above, and it will be found that fox-glove often applies itself to these cases with happy effect. I am now speaking of adult affections, and the average dose of the medicine I am accustomed to order is from ten to fifteen drops of the saturated tincture two or three times a day. In these instances we have the arterial power, or rather irritation, lessened, while a general tone is at the same time imparted; and it is a very curious fact, that digitalis, when it does do good in these chronic irritations, always improves the pulse by improving the tone of the arteries." 197.

From the Doctor's remarks on epilepsy, hypochondriasis, and several other affections connected with or leading to mania—together with his cursory observations on the usual remedies employed, we do not see any thing either very new or very striking to extract. We must therefore close our notice of the work in the recapitulatory words of the author himself.

" 'Madness is a term that means every thing, and means nothing.' Although we admit its essentials to consist in a vivid conception, to the extent of actual belief in unreal things, or the exaltation of the fancy into supposed perception; as it must at the same time be admitted, that delusion, more or less substantial and confirmed, is the occasional fate of us all, it follows, that the difference between sanity, and acknowledged insanity, is rather a difference in degree, than in kind.

Or take it in another way. 'Madness is a disease of motives;' the nervous invalid who is not susceptible of sane impulse, who cannot rise from her chair and pace the room, who is incapable, for the present, of feeling for her family as she was wont to feel, merely requires her susceptibility to common motives to be reproduced, or such an application of the motives on the part of others, or as the result of incident, that they shall act with sufficient force to subdue the insensibility or inaptitude, and the malady is cured. Incapacity, was therefore, in her case, both real and ideal; but she was, in point of fact, as mad as the loudest bawler in a solitary cell of a lunatic asylum." 227.

Dr. Uwins is, unquestionably a judicious and experienced physician, very cautious of all extremes, both in theory and practice—and appearing always to keep in view the wise maxim—" in medio tutissimus ibis." But this very caution and balancing between extremes, produces a degree of wavering and incertitude in Dr. Uwins' writings, which leave a doubt upon every subject, in the mind of the reader—especially of the inexperienced.

